# A rare case of primary peripheral epithelial myoepithelial carcinoma of lung: Case report and literature review

**DOI:** 10.1097/MD.0000000000004371

**Published:** 2016-09-02

**Authors:** Cheng Shen, Xin Wang, Guowei Che

**Affiliations:** Department of Thoracic Surgery, West China Hospital, Sichuan University, Chengdu, China.

**Keywords:** computed tomography, epithelial myoepithelial carcinoma, pathology, surgery

## Abstract

**Background:**

Primary salivary gland–type tumors of lung are rare. Epithelial–myoepithelial carcinoma (EMC) of the lung is a minor salivary gland–type tumor subtype.

**Methods:**

We report a very rare case of EMC located in the peripheral left lower lobe that was diagnosed in a 58-year-old man and this is the first study in which we summarize all the patients with primary peripheral lung EMC concerned with the clinical features. Informed consent was obtained from the patient.

**Results:**

Chest computed tomography displayed an anomalous soft tissue mass with slightly lobular borders in the peripheral segment of the left lower lobe and closed to the visceral pleura. The surgery was performed by using video-assisted thoracic surgery. Grossly, the tumor was solitary, well-circumscribed, and unencapsulated endobronchial lesion. A microscopic examination revealed that it was circumscribed, although the tumor borders may show single cells or clusters of cells proliferating away from the main tumor mass. The inner tubular layer showed epithelial cell characteristics, whereas the outer layer exhibited myoepithelial cell characteristics. Immunostaining for P40, P63, and cytokeratin 5/6 was positive. However, the anaplastic lymphoma kinase-V, thyroid transcription factor-1, synaptophysin, chromogranin A and napsin A were negative.

**Conclusions:**

Literature review showed that most of patients with peripheral EMC were asymptomatic. Computed tomography and magnetic resonance imaging scans are able to indicate the presence of peripheral EMC. Pathological analysis is an effective method to clarify the diagnosis. Surgery is a regular treatment method. To facilitate the preoperative diagnosis and avoid the misdiagnosis of such a rare disease, more cases will need to be reported.

## 1 Introduction

Primary salivary gland–type tumors (SGTTs) of lung are rare, accounting for 0.1% to 0.2% of all lung tumors.^[1]^ Common SGTT subtypes include mucoepidermoid carcinoma and adenoid cystic carcinoma. Epithelial myoepithelial carcinoma (EMC) of the lung is a minor SGTT subtype. About 120 cases have been reported in the world literature, most of which were located in salivary glands, except for a few cases occurring in unusual locations such as trachea and bronchus.^[2–4]^ EMC is pathologically characterized by a dual cell population, including an inner layer of cuboidal epithelial cells that are peripherally bounded by a layer of myoepithelial cells.^[5,6]^ Herein, we report a very rare case of EMC located in the peripheral left lower lobe that was diagnosed in a 58-year-old man and this is the first study in which we summarize all the patients with primary peripheral lung EMC concerned with the clinical features.

## 2 Case report

**Figure 1:**
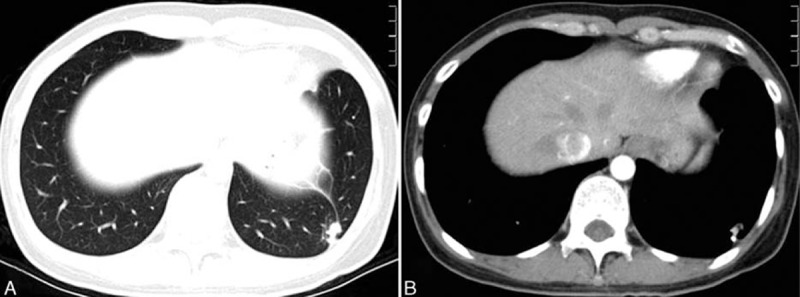
(A and B) An anomalous soft tissue mass with slightly lobular borders in the peripheral segment of the left lower lobe and closed to the visceral pleura.

A 58-year-old man was referred to our hospital for a routine health check. He denied symptoms, including chest pain, cough, and dyspnea. He was a nonsmoker. Physical examination revealed normal breathing sounds in both lung fields. Laboratory findings were within normal limits. His pulmonary function tests revealed normal performance. Chest computed tomography (CT) displayed an anomalous soft tissue mass with slightly lobular borders in the peripheral segment of the left lower lobe and closed to the visceral pleura (Fig. 1A and B). The mass measured 1.2 cm diametrically. There was no evidence of bronchial or vascular invasion. The bronchoscopic examination showed nothing in trachea and bronchus. The patient, however, refused to have the CT-guided percutaneous aspiration examination.

**Figure 2:**
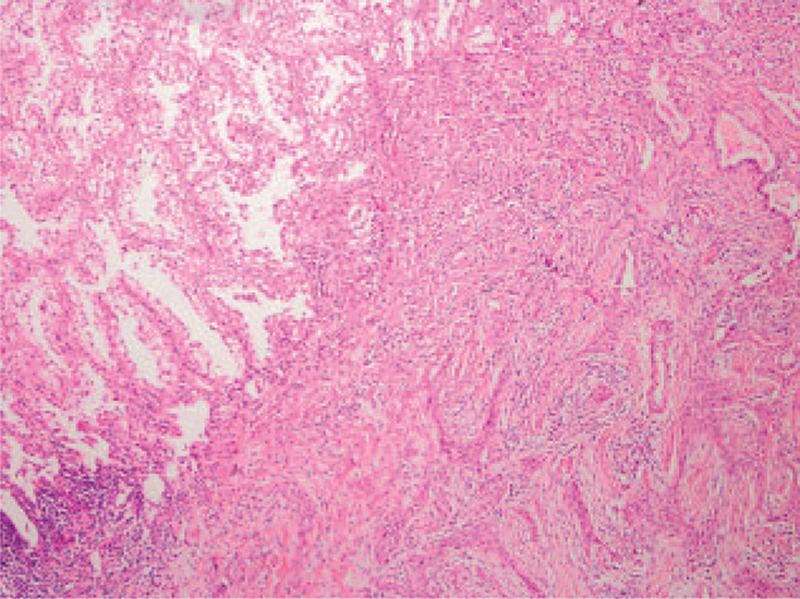
H&E staining of resected lung tissue (×100). H&E = hematoxylin-eosin staining.

**Figure 3:**
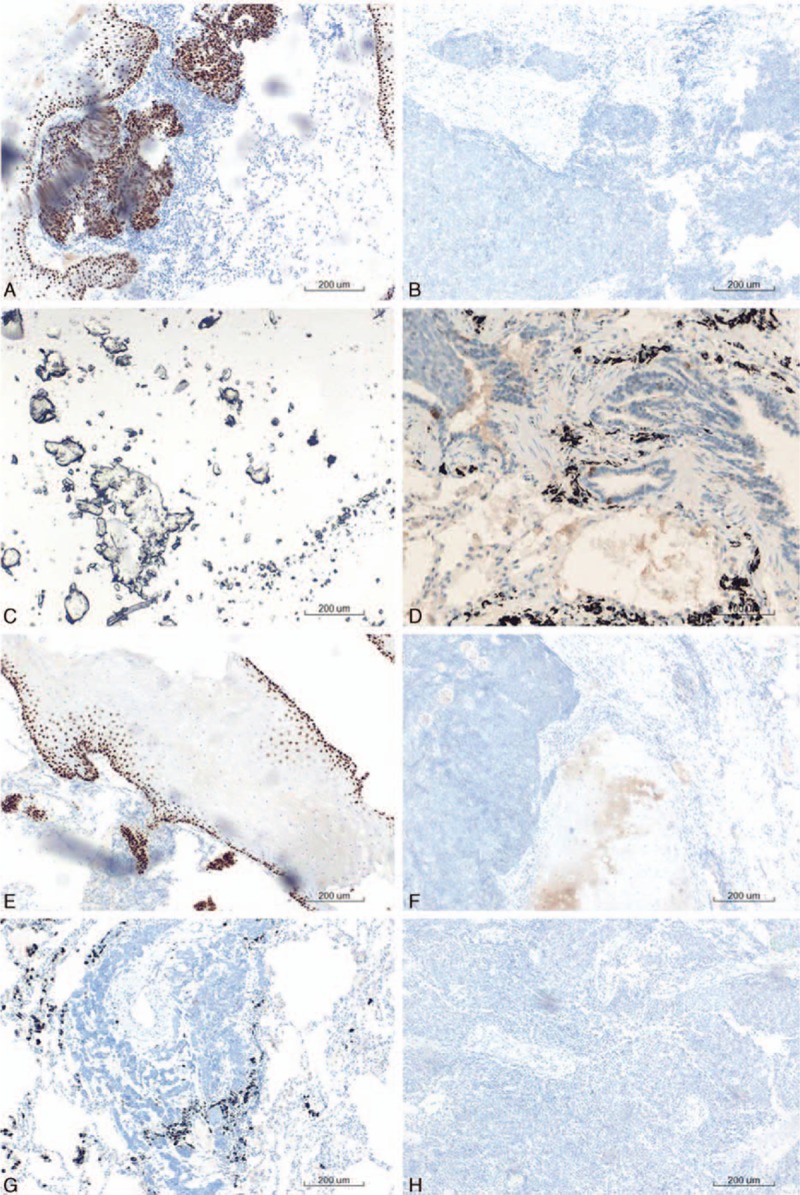
(A) Immunoreactivity in epithelial cells for P40 (×200); (B) immunoreactivity in epithelial cells for ALK (×200); (C) immunoreactivity in epithelial cells for CK5/6 (×200); (D) immunoreactivity in epithelial cells for Syn (×200); (E) immunoreactivity in epithelial cells for P633 (×200); (F) immunoreactivity in epithelial cells for TTF-1 (×200); (G) immunoreactivity in epithelial cells for CgA (×200); (H) immunoreactivity in epithelial cells for napsin A (×200). ALK = anaplastic lymphoma kinase, CgA = chromogranin A, CK = cytokeratin, Syn = synaptophysin, TTF-1 = thyroid transcription factor-1.

As a diagnosis was not established, surgery was scheduled. We approached the tumor by using video-assisted thoracic surgery (VATS). The surgery was performed in the lateral decubitus position. Grossly, the tumor was solitary, well-circumscribed, and unencapsulated endobronchial lesion, measuring 1.3 × 1.1 × 1.2 cm. A microscopic examination revealed that it was circumscribed, although the tumor borders may show single cells or clusters of cells proliferating away from the main tumor mass (Fig. 2). The inner tubular layer showed epithelial cell characteristics, whereas the outer layer exhibited myoepithelial cell characteristics. Immunostaining for P40, P63, and cytokeratin 5/6 was positive. However, the anaplastic lymphoma kinase-V, thyroid transcription factor-1 (TTF-1), synaptophysin, chromogranin A, and napsin A were negative (Fig. 3). The postoperative course was ordinary. The patient was discharged 3 days after the operation with no complication. He has been followed up for 8 months without evidence of recurrence.

## 3 Discussion

Epithelial–myoepithelial tumors are rare neoplasms that occur more commonly in salivary glands, where they represent approximately 0.5% of primary tumors.^[7]^ The presence of EMC in the salivary gland was first described by Donath et al in 1972.^[8]^ The tracheobronchial glands are considered to be counterparts of the minor salivary glands in the respiratory tract and can develop similar tumors. Within this group of neoplasia EMC of the respiratory tract is very rare and the diagnosis is often difficult.^[2]^ It has occurred in patients aged between 8 and 103 years with a mean age about 60 years and displayed a female predominance (female to male ratio = 2:1).^[9]^ This tumor does not seem to be related to cigarette smoking.^[4,10]^ As seen in Table 1, among all the patients, there are only 4 female patients and male to female ratio is 4:1. The mean age is 57.4 years and 5 patients are smokers. The symptoms are varied, ranging from asymptomatic cases, as our summary and case showed, to cough, thoracic pain, dyspnea, abdominal pain, or shortness of breath in all patients.

**Table 1:**
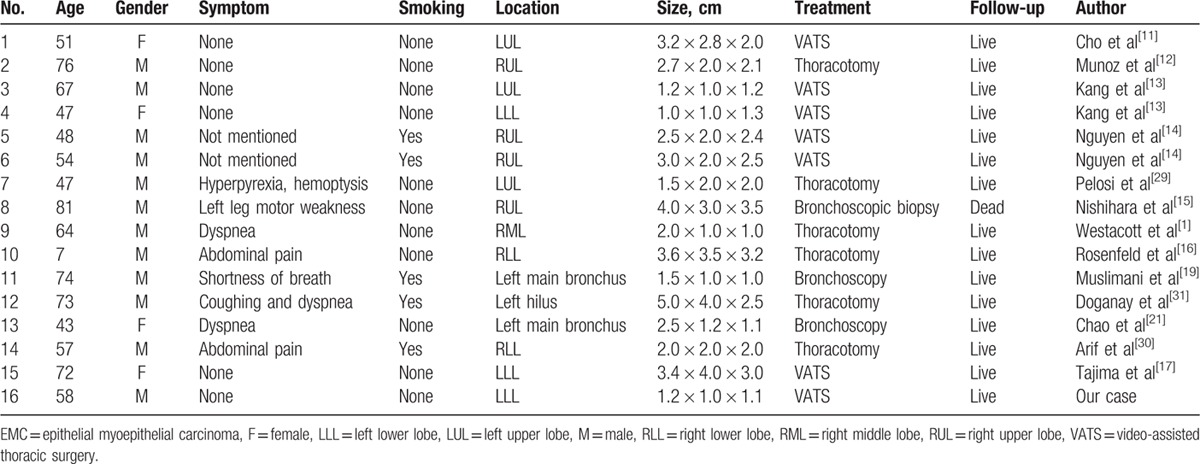
Characteristics of patients with pulmonary EMC.

As these tumors are rare, criteria for diagnosing EAC radiologically do not exist. EMC can be dependably diagnosed by CT, magnetic resonance imaging (MRI), and positron emission tomography–CT. Some reports showed that the margin of the mass was relatively well defined, and the internal CT density indicated a mixture of cystic and solid components.^[2,18]^ On MRI, the T1-weighted sequence showed the mass mostly with isosignal intensity to muscle tissue and partially with slightly higher signal intensity. The T2-weighted sequence of the mass showed heterogeneous high signal intensity, along with clear visualization of the septa and capsule-like structures. Contrast-enhanced MRI showed strong heterogeneous enhancement of the mass.^[19]^ Zhu et al reported that positron emission tomography–CT images show an oval nodule with slight but obvious increased (18)F-fluoro-2-deoxy-d-glucose uptake in the lobe.^[20]^ Most of the previously reported cases of pulmonary EMC have formed intraluminal polypoid masses occluding the lumen of the bronchus and they can be found by fiber-optic bronchoscopy. There are 6 tumors located in pulmonary parenchyma in our summary (cases 1–6). The patient we present in this report is the seventh reported case of a tumor located in the left lower peripheral pulmonary parenchyma. In this case, fiber-optic bronchoscopy examination is normal and chest CT displayed an anomalous soft tissue mass with slightly lobular borders in the peripheral segment of the left lower lobe and closed to the visceral pleura.

In most reviewed cases, an evident relation and connection with the bronchial tree is observed.^[18,21]^ However, in this case, the tumor presents as an intraparenchymatous mass without apparent connection with bronchus. Its size varies, ranging from 1 to 16 cm in diameter.^[22–24]^ Microscopically, it is a multinodular lesion presenting a bicellular pattern of proliferating epithelial cells with eosinophilic centrally located nucleus and myoepithelial cells with abundant clear cytoplasm.^[7]^ Immunohistochemically, the epithelial component is positive for keratin and carcino-embryonic antigen (CEA), while myoepithelial component is positive for S-100, p63, and CD10. Epithelial cells are positive for TTF-1, unlike myoepithelial cells.^[25]^ As seen in our case, P40, P63, and cytokeratin 5/6 were positive and the anaplastic lymphoma kinase-V, TTF-1, synaptophysin, chromogranin A, and napsin A were negative.

The differential diagnosis of this disease includes pleomorphic adenomas and adenoid cystic carcinomas. Pleomorphic adenoma is a benign, slow-growing tumor, most commonly of the salivary gland, occurring as a small, painless, firm nodule, usually of the parotid gland, but also found in any major or accessory salivary gland anywhere in the oral cavity.^[23]^ Adenoid cystic carcinoma is characterized by bands or cylinders of hyalinized or mucinous stroma separating or surrounded by nests or cords of small epithelial cells. When the cylinders occur within masses of epithelial cells, they give the tissue a perforated, sieve-like, or cribriform appearance. Such tumors occur in the mammary glands, the mucous glands of the upper and lower respiratory tract, and the salivary glands. They are malignant but slow-growing, and tend to spread locally via the nerves.^[26]^

Although a variety of names have been attached to these tumors, the term EMC of the bronchus is preferred because these tumors are potentially malignant, despite the majority behaving in an indolent fashion. Malignant EMC of the lung has been debated by many authors, and Wilson and Moran suggested that this entity be identified as a carcinoma to convey the malignant potential of these tumors to clinicians and patients.^[27]^ Their study indicated that the tumors could have malignant potential, based on the malignant behavior of EMC of the salivary gland. EMC of the salivary gland demonstrated a local recurrence rate of 23% to 80% and a 14% to 25% rate of metastasis.^[28]^ The biological behavior of pulmonary EMC is still unclear. Pelosi et al^[29]^ found out that the protein p27/kip-1 is a cyclin-dependent kinase inhibitor that blocks cell cycle in G0 and G1. Arif et al^[30]^ showed the direct in vivo evidence that p27/kip-1 also functions as an oncogene. An abnormal subcellular location of p27/kip-1 into the myoepithelial cell would provoke the loss of its growth inhibition function through the lack of restriction of proliferation of myoepithelial component.^[29]^

Although optimal therapy for pulmonary EMC has yet to be defined, a clinical course similar to that of salivary EMC may be expected. However, one of the characteristic features of salivary gland EMC is the long interval between diagnosis and recurrence and metastasis. Considering the similar histopathological features of pulmonary EMC and salivary EMC, recurrence and metastasis can also be expected in pulmonary EMC in the long term. It appears that lobectomy or pneumonectomy and sometimes sleeve resection may be the treatment of choice. The follow-up periods of the reported cases range from 6 to 36 months.^[31]^ None of the reported cases had evidence of disease during their follow-up periods. The patient we describe is still alive without recurrence or metastasis 8 months after undergoing pneumonectomy by VATS. VATS has become a widely accepted alternative for the resection of thorax tumors. Compared with open thoracotomy, this minimally invasive procedure was related with many advantages, such as less trauma and faster recovery. With the development of thoracoscopic instruments and techniques, the application of uniportal VATS has become the regular method, especially for the peripheral pulmonary neoplasm. Adjuvant radiotherapy is also required in the case of 4-cm or larger primary tumors or positive surgical margins.^[32]^ Chemotherapy is used in patients with advanced disease or surgically unresectable disease, with various agents alone or in combination (cisplatin, cyclophosphamide, doxorubicin, mitoxantrone, carboplatin, and vinorelbine).^[32]^

## 4 Conclusions

Primary lung tumors mimicking the salivary gland–type neoplasms are extremely rare. Primary peripheral EMC is an uncommon tumor in this group, and only 6 cases have been reported. Most of patients with peripheral EMC were asymptomatic in our summary. CT and MRI scans are able to indicate the presence of peripheral EMC. Pathological analysis is an effective method to clarify the diagnosis. Surgery is a regular treatment method. To facilitate the preoperative diagnosis and avoid the misdiagnosis of such a rare disease, more cases will need to be reported.
